# Subgrouping siblings of people with autism: Identifying the broader autism phenotype

**DOI:** 10.1002/aur.1544

**Published:** 2015-09-02

**Authors:** Emily Ruzich, Carrie Allison, Paula Smith, Peter Watson, Bonnie Auyeung, Howard Ring, Simon Baron‐Cohen

**Affiliations:** ^1^ Department of PsychiatryAutism Research Centre, University of CambridgeDouglas House, 18B Trumpington RoadCambridgeCB2 8AHUK; ^2^ Department of PsychiatryCambridge Intellectual and Developmental Disabilities Research Group, University of CambridgeDouglas House, 18B Trumpington RoadCambridgeCB2 8AHUK; ^3^ NIHR CLAHRC for the East of EnglandDouglas House, 18B Trumpington RoadCambridgeCB2 8AHUK; ^4^ Cambridgeshire and Peterborough NHS Foundation TrustPeterboroughCB21 5EFUK; ^5^ CLASS Clinic, Cambridgeshire and Peterborough NHS Foundation TrustPeterboroughCB21 5EFUK; ^6^ MRC Cognition and Brain Sciences Unit15 Chaucer RoadCambridgeCB2 7EFUK; ^7^ Department of PsychologySchool of Philosophy, Psychology and Language Sciences, University of Edinburgh7 George SquareEdinburghEH9 1JDUK

**Keywords:** autism, Autism‐Spectrum Quotient, autistic traits, siblings, sex differences, broader autism phenotype

## Abstract

We investigate the broader autism phenotype (BAP) in siblings of individuals with autism spectrum conditions (ASC). Autistic traits were measured in typical controls (*n* = 2,000), siblings (*n* = 496), and volunteers with ASC (*n* = 2,322) using the Autism‐Spectrum Quotient (AQ), both self‐report and parent‐report versions. Using cluster analysis of AQ subscale scores, two sibling subgroups were identified for both males and females: a cluster of low‐scorers and a cluster of high‐scorers. Results show that while siblings as a group have intermediate levels of autistic traits compared to control individuals and participants with ASC, when examined on a cluster level, the low‐scoring sibling group is more similar to typical controls while the high‐scoring group is more similar to the ASC clinical group. Further investigation into the underlying genetic and epigenetic characteristics of these two subgroups will be informative in understanding autistic traits, both within the general population and in relation to those with a clinical diagnosis. ***Autism Res*** *2016, 9: 658–665*. © 2015 The Authors Autism Research published by Wiley Periodicals, Inc. on behalf of International Society for Autism Research

## Introduction

Autistic traits lie along a continuum from those with a clinical diagnosis of autism spectrum conditions (ASC) into the general population. This is due to the genetic and epigenetic nature of ASC, where multiple candidate loci contribute to the inheritance of autism [Persico & Bourgeron, 2006]. While ASC itself is complex and heterogeneous, familial and genetic studies of autistic traits indicate these too are partly heritable [Abrahams & Geschwind, 2008; Chakrabarti et al., 2009; Folstein & Rosen‐Sheidley, 2001; Freitag, 2008]. Family studies find phenotypic links to autistic traits not only in twins [Le Couteur et al., 1996; Taniai, Nishiyama, Miyachi, Imaeda, & Sumi, 2008] but also in other first‐degree relatives such as siblings and parents [Holt et al., 2014; Sucksmith, Roth, & Hoekstra, 2011; Wheelwright, Auyeung, Allison, & Baron‐Cohen, 2010; Zhao et al., 2007]. Even second‐degree relatives show evidence of traits relevant to an aptitude with objects and systems: both maternal and paternal grandfathers of children with autism are over‐represented in systemizing professions such as science, engineering, and physics, compared to the grandparents of typically developing children or children with other neurodevelopmental conditions [Baron‐Cohen et al., 1998; Baron‐Cohen, Wheelwright, Stott, Bolton, & Goodyer, 1997; Roelfsema et al., 2012]. This collection of traits has been termed the extended or broader autism phenotype (BAP).

As with parents and children, siblings share approximately 50% of their genetic makeup with their brothers or sisters, often in addition to a similar environment during childhood. Younger siblings of children with autism have an elevated chance of developing the condition themselves: in 1994, when the rate of ASC in the general population was estimated to be 1 per 1,000, recurrence rates in siblings of those with ASC were estimated at 3%, while between 10 and 20% of siblings exhibited what at the time was termed a “lesser variant” of autism [Bolton et al., 1994]. A report in 2010, when rates of ASC were estimated to be 1% of the general population [Baron‐Cohen, Scott, et al., 2009], indicated the rate of recurrence among siblings to be 10.9% [Constantino, Zhang, Frazier, Abbacchi, & Law, 2010]. By 2013, the rate of ASC in siblings had risen to 1 in 3 [Gronborg, Schendel, & Parner, 2013; Ozonoff et al., 2011]. In adults, there is nearly a 1‐in‐three chance of having one or more features of the extended autism phenotype if one has a sibling with autism [Folstein & Rosen‐Sheidley, 2001].

The Autism‐Spectrum Quotient (AQ), a 50‐item measure for characterizing autistic traits in individuals with average IQ or above, is informative as it contains five subscales covering a variety of traits known to be affected in people with a diagnosis of ASC and in individuals with the BAP: communication, social skills, imagination, attention to detail, and attention switching [Baron‐Cohen, Wheelwright, Skinner, Martin, & Clubley, 2001]. AQ score exhibits a continuous quasi‐normal distribution in several studies in male and female individuals with ASC [Auyeung, Baron‐Cohen, Wheelwright, & Allison, 2008; Baron‐Cohen et al., 2014; Wheelwright et al., 2006], other clinical conditions such as anxiety and obsessive compulsive disorder [Hoekstra, Bartels, Cath, & Boomsma, 2008], particular professions such as those in the sciences [Wheelwright et al., 2006], and the general population [Ruzich et al., 2015]. Of particular relevance is a study [Wheelwright et al., 2010] which found higher AQ scores in parents of children with ASC, compared to parents of typically developing children; and a study which reports similar findings in siblings [Ruzich et al., submitted].

In addition to finding that male and female adult siblings, AQ scores fall between cases and controls (as well as female child and female adolescent siblings), Ruzich et al. [submitted] found a bimodal distribution of AQ scores in sibling groups. This bimodal distribution hints at the possibility that there may be distinct sibling subgroups, defined by behavioral differences. The current study aims to better understand the distribution of autistic traits in a sibling cohort. We address two questions: first, are there subgroups within the siblings, defined by their AQ responses? Second, do any of these subgroups fall more clearly within the bounds of the BAP? Identifying phenotypic subgroups may be informative at a genetic level, even if there is no clinical utility in defining such boundaries. We conducted a hierarchical cluster analysis of AQ subscale scores akin to the method carried out in individuals with ASC [Ring, Woodbury‐Smith, Watson, Wheelwright, & Baron‐Cohen, 2008] to answer the first question, and then explored the relation of any subgroup clusters to the previously described BAP.

## Methods

### Instrument

Details about the instructions for administration and scoring of the various versions of the AQ can be found elsewhere [Ruzich et al., submitted]. The AQ consists of 50 items, divided into 5 subscales consisting of 10 items each. It assesses domains of cognitive strengths and difficulties related to ASC. The AQ (adult version) is designed as a self‐report measure, while the Adolescent and Child versions are to be completed by a parent or guardian of the individual in question [Auyeung et al., 2008; Baron‐Cohen, Hoekstra, Knickmeyer, & Wheelwright, 2006; Baron‐Cohen et al., 2001].

### Participants

Three groups of participants were selected: individuals with a clinical diagnosis of ASC, individuals with no personal history of ASC but with a sibling with a diagnosis (hereafter referred to as the sibling group), and individuals with no personal or family history of ASC (controls). Within each group, three cohorts were distinguished: those who had taken the 50‐item AQ self‐report, the AQ‐Adolescent parent‐report, and the AQ‐Child parent‐report.

#### ASC cases

Data from individuals with a clinical diagnosis of ASC were collected from the Cambridge autism research database (CARD). Here, volunteers (or their parents/caregivers) can register online (www.autismresearchcentre.com) and provide details about their diagnosis and complete an online version of the AQ. Only individuals who provided full diagnostic information (name of clinic, name of clinical psychologist or psychiatrist, and date of diagnosis) and completed the AQ test were included in the analysis.

#### Siblings

Data from siblings of individuals with ASC were also collected from CARD. Parents of children and adolescents were recruited and invited to respond on behalf of each of their children, regardless of diagnosis. For adults, volunteers were invited by their brother or sister with ASC via an online form (www.autismresearchcentre.com); a minority of adults from the general population interested in taking part in psychology research also were recruited online at a site seeking individuals from the general population (www.cambridgepsychology.com). For this group, individuals were selected if they had co‐registered with a sibling with ASC or if they had reported having a sibling with ASC, even if the sibling had not registered online. Individuals were excluded if they reported a suspected diagnosis or that they were seeking a diagnosis but had not yet obtained one.

#### Controls

Data from individuals with no personal or family history of ASC were collected from the SCORE cohort [Allison et al., 2007; Baron‐Cohen, Auyeung, et al., 2009]. In the SCORE study, questionnaires were distributed via schools in the Cambridgeshire area to parents/caregivers and their children (initially recruited aged 5–9 years old; re‐contacted for AQ data when aged 6–11) and were returned to researchers by Freepost envelope.

### Processing

Data were imported into R [RCoreTeam, 2014], cleaned of incomplete AQ tests and exclusions were made if the participant was older or younger than the age range recommended for each test (AQ‐Child 4–11 years; AQ‐Adolescent 12–16 years; AQ 16+ years). Individuals duplicated between age groups (e.g., an individual whose parent had completed the AQ‐Child and then later had completed the AQ‐Adolescent) were randomly selected for inclusion in just one of the measures using a process that maximized each group's sample size, and multiple members of a family were removed (e.g., if two siblings of an individual with autism had taken the AQ, only one was included for analysis using an automated and randomized selection procedure). Individuals with null scores or missing data were also removed. Equal numbers of males and females were included via a random selection process.

Due to previous findings indicating similar distributions of scores for parent‐report versions of the AQ [Ruzich et al, submitted], the AQ‐Adolescent and AQ‐Child were combined into one category, termed here AQ Parent‐Reports (P‐R). Details of the participants can be found in Table 1.

**Table 1:** Total *N* Participants

	ASC	Siblings	Controls
AQ	1,794	138	688
AQ P‐R	528	358	1312

Participant groups are 50% female.

### Procedure

Data were analyzed in R [RCoreTeam, 2014]. First, a cluster analysis was performed on the subscale scores to identify potential subgroups within the sibling cohorts for males and females. Hierarchical clustering [Ward, 1963] was first applied, and then flat clustering (k‐means) was used as confirmation of cluster group membership. Hierarchical clustering uses a measure of dissimilarity to assign group membership to individuals or to separate a group into smaller clusters. Ward's method agglomerates clusters through minimizing variance. Partitional or divisive k‐means clustering was also used to confirm cluster assignment, but the Ward solution was used for final analysis, as k‐means clustering assumes that clusters will be equally sized and convex in shape. Agreement between different clustering methods was evaluated using percentage agreement in a two‐way table (clusters assigned by one method as rows and clusters assigned by the other method as columns). Dendrograms were used to determine optimal clusters.

Next, the groups were examined to determine the respective compositions of the participant groups as relating to the BAP. AQ distribution was divided based on standard deviations from the mean of the control data [Wheelwright et al., 2010]. In this method, broad, medium, and narrow autism phenotype (BAP, MAP, and NAP) were calculated from the scores of the control males and females as follows: BAP is defined as AQ scores of 1 to 2 SDs above the mean. MAP is defined as AQ scores of 2–3 SDs above the mean. NAP is defined as AQ scores ≥ 3 SDs above the mean.

## Results

### Sibling Subgroups

**Figure 1:**
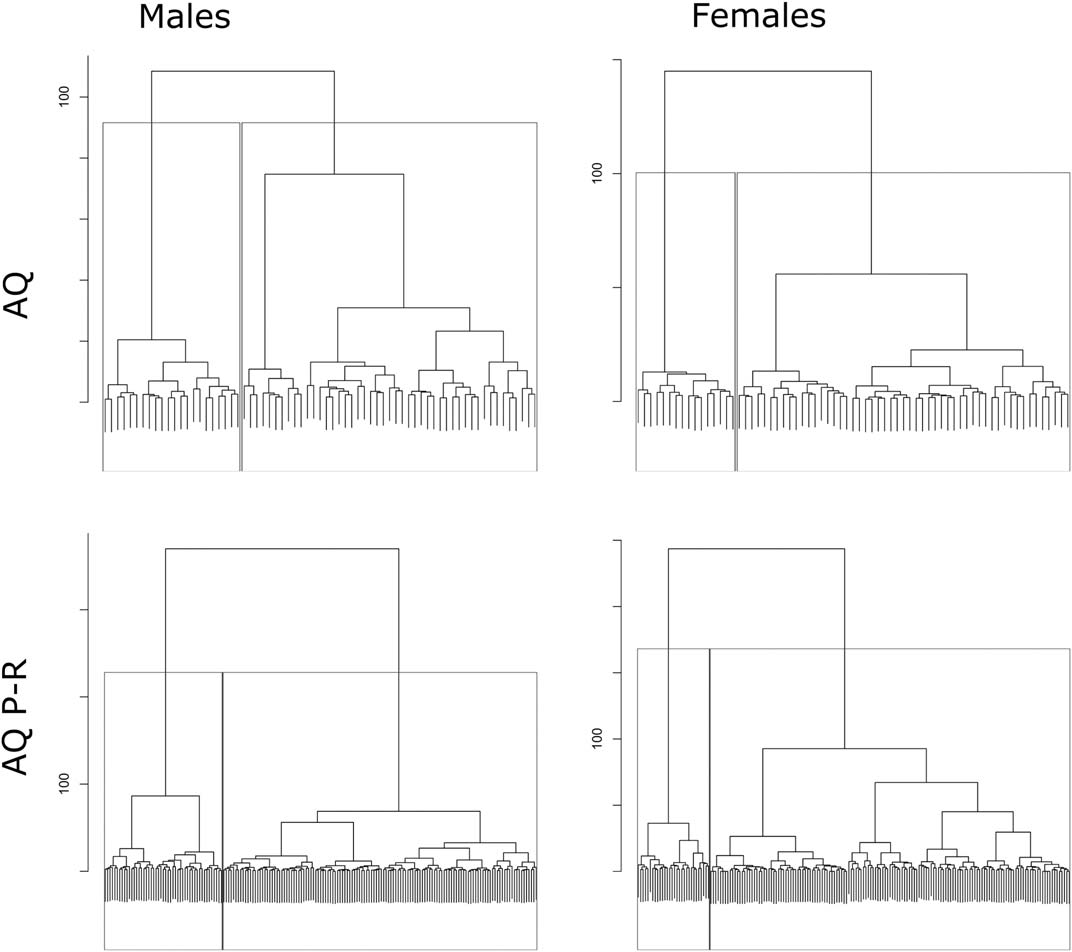
Ward hierarchical cluster dendrograms. Stepwise cluster assignment of individuals represented by bottom‐up tree structure; cluster iterations indicated on *y*‐axis. Cluster selections stopped at n‐2 (indicated by boxes).

Due to the shape of the sibling distribution described elsewhere [Ruzich et al., submitted], a 2‐cluster model was selected for exploratory analysis of the data. This solution was further supported by silhouette analysis of n‐cluster solutions, where a 2‐cluster model was preferred due to a large positive silhouette, similarly sized cluster silhouettes, and an absence of clusters falling below the average width. Both Ward agglomerative hierarchical clustering and k‐means partitional clustering were used to divide the sibling cohorts into subgroups; however, k‐means was only used as a confirmatory measure, as it was unknown if clusters would appear convex and of equal size. Cluster hierarchy plots are shown in Figure 1. Percentage of correspondence between hierarchical and k‐means methods was calculated; for 2‐cluster models, the male AQ P‐R had 93.3% agreement, female AQ P‐R had 93.3% agreement, male AQ self‐report had 62.9% agreement, and female AQ self‐report had 95.7% agreement.

Descriptive statistics (n individuals, means, and standard deviations) for resultant clusters are found in Table 2. The data indicate for all cases that Cluster 1 is globally low across all subscale scores while Cluster 2 is high. Between‐cluster t‐tests were significant for all subscales, though differences were least pronounced in the Attention to Detail subscale (P values approaching 0.01). This demonstrates that autistic traits as measured by the AQ vary together to create a high‐ and a low‐scoring group on all subscales, rather than clusters that are predicted by one or two subscale scores alone.

**Table 2:** Means and Standard Deviations for Male and Female Cluster AQ Subscale Scores

		Males		Females	
		Cluster 1 (N = 22)	Cluster 2 (N = 47)	Cluster 1 (N = 53)	Cluster 2 (N = 16)
AQ	Communication	0.91 (1.15)*	4.57 (2.48)	2.15 (1.66)*	7.06 (1.44)
	Social Skills	0.73 (0.63)*	5.09 (2.92)	2.13 (1.97)*	8.25 (1.34)
	Imagination	1.36 (1.22)*	3.85 (2.64)	1.62 (1.61)*	5.50 (1.83)
	Attention to Detail	3.50 (2.32)*	5.72 (2.21)	4.43 (2.07)*	6.94 (2.29)
	Attention Switching	2.50 (1.71)*	6.53 (2.00)	3.89 (2.07)*	8.13 (1.71)

		Cluster 1 (N = 130)	Cluster 2 (N = 49)	Cluster 1 (N = 149)	Cluster 2 (N = 30)
AQ P‐R	Communication	1.62 (1.48)*	6.59 (2.87)	1.56 (1.51)*	6.40 (1.98)
	Social Skills	0.95 (1.07)*	6.33 (2.36)	0.95 (1.19)*	6.17 (1.42)
	Imagination	1.50 (1.14)*	5.12 (2.34)	0.81 (1.14)*	3.80 (2.34)
	Attention to Detail	3.42 (2.22)*	6.31 (2.59)	3.66 (2.23)*	5.13 (2.34)
	Attention Switching	2.65 (1.71)*	7.20 (2.25)	2.58 (1.94)*	6.60 (1.87)

Differences between cluster subscale scores were evaluated

* *P* < 0.01

The derived low‐scoring and high‐scoring sibling clusters were then compared with groups of individuals with ASC and control participants for males and females who had taken the AQ and AQ P‐R to examine these in relation to the broader autism phenotype.

### Broader Autism Phenotype

BAP, MAP, and NAP, along with average‐ (within 1 SD of the mean) and low‐scorers (below 1 SD of the mean), were calculated for each group using the mean and standard deviation of the relevant control group (Table 3).

**Table 3:** Cutoff Scores

		Low	Average	BAP	MAP	NAP
AQ	Males	<10.73	10.73 ≥ AQ < 24.52	24.52 ≥ AQ < 31.41	31.41 ≥ AQ < 38.31	≥ 38.31
	Females	<6.65	6.65 ≥ AQ < 19.14	19.14 ≥ AQ < 25.39	25.39 ≥ AQ < 31.64	≥ 31.64
AQ P‐R	Males	<7.22	7.22 ≥ AQ < 24.48	24.48 ≥ AQ < 33.11	33.11 ≥ AQ < 41.74	≥ 41.74
	Females	<6.15	6.15 ≥ AQ < 18.76	18.76 ≥ AQ < 25.07	25.07 ≥ AQ < 31.38	≥ 31.38

Calculated based on respective control group mean and standard deviation: for males, AQ mean = 17.62 (SD = 6.90) AQ P‐R mean = 15.85 (8.63); in females, AQ mean = 12.90 (SD = 6.25) AQ P‐R mean = 12.45 (6.31).

**Figure 2:**
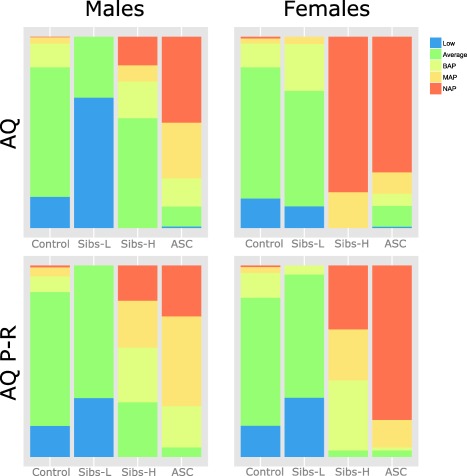
BAP/MAP/NAP distributions. BAP is defined as AQ scores of 1 to 2 SDs above the mean. MAP is defined as AQ scores of 2–3 SDs above the mean. NAP is defined as AQ scores ≥3 SDs above the mean.

By scaling the data of all groups to the control scores in this way (as opposed to using the 50‐point AQ scale as an absolute) we hoped to reveal more about the sibling groups in relation to controls and to individuals with autism. The relative percentage of individuals within each group falling within each band is presented in Figure 2. Examination of this Figure shows that, as before, the ASC groups have the highest percentage of individuals with the extended autism phenotype (defined here as the combined BAP/MAP/NAP), followed by high‐scoring siblings, while low‐scoring sibling and control groups have relatively few individuals in this range. Extended phenotype (EP) and low phenotype (LP) bands were evaluated using chi‐squared comparisons between controls and low‐scoring siblings, between ASC and high‐scoring siblings, and between the two sibling groups (Table 4).

**Table 4:** LP and EP χ^2^ Values

		Controls vs low‐scoring		Low‐scoring vs high‐scoring		High‐scoring vs ASC	
AQ Version	Sex	LP	EP	LP	EP	LP	EP
AQ	Males	16.28*	12.05*	68.18*	42.55*	65.88*	88.74*
	Females	0.62	3.42	11.32*	40.07	0.67	0.71
AQ P‐R	Males	16.16*	38.84*	30.77*	71.43*	30.77*	95.08*
	Females	16.31*	56.28*	30.87*	83.44*	30.87*	83.37*

Chi‐squared tests were calculated for controls and low‐scoring siblings, ASC and high‐scoring siblings, and between the two sibling groups for upper and lower bands.

* *P* < 0.001

When assessing the proportions of individuals in each sibling cluster that fall into the EP or the LP, low‐scoring siblings have an even greater proportion of individuals in the LP band than do controls, while high‐scoring siblings have comparable numbers of individuals in the EP band, compared to individuals with ASC. The exception to this was that for adult females, low‐scoring siblings did not differ from control participants and high‐scoring siblings did not differ from ASC participants in both the low and extended phenotype bands.

## Discussion

The current analysis of AQ scores for siblings of individuals with ASC indicates that subgroups exist for males and females taking self‐ and parent‐reports, and that these subgroups encapsulate distinct behavioural patterns of autistic traits. For males and females, and for self‐ and parent‐report AQ versions, a two‐cluster model fits the data where individuals have membership either to cluster 1, which includes siblings more closely resembling people without ASC, or cluster 2, which comprises people with elevated autistic traits. These models are reliable as they are reproduced with a high degree of accuracy, by both hierarchical and k‐means methods: only for adult males was percentage correspondence less than 90%. For all females, and for male children, the low‐scoring cluster contained a greater proportion of individuals than the high‐scoring cluster, though for adult males, this trend was reversed (see Fig. 1 and Table 2). It is noted that the clusters were globally high or globally low, rather than having a subset of AQ subscale scores standing out as predictors (Table 2). In the field of autism research, there is ongoing debate as to whether the spectrum represents aetiologically distinct conditions with varying symptom profiles or a unified profile with varying severity [Constantino et al., 2004; Folstein & Rosen‐Sheidley, 2001; Lau, Kelly, & Peterson, 2013; Lenroot & Yeung, 2013; Ronald et al., 2006; Spiker, Lotspeich, Dimiceli, Myers, & Risch, 2002]. This result for sibling groups with no sub‐scale spikes adds to this issue from a nonclinical perspective, and corresponds to the similar observation that, in individuals with autism, there is a continuous severity gradient as measured by the AQ [Ring et al., 2008].

When assessing whether either one or both of the high or low‐scoring sibling clusters can be considered to fall into the extended phenotype, we used control participant scores to rescale sibling and ASC scores. By examining the resulting high (extended phenotype) and low (low phenotype) bands, control individuals are statistically different from low‐scoring individuals. Using this scaling method, low‐scoring siblings are shifted to be “less autistic” than the full range of controls, except for in adult females. Interestingly, this BAP analysis reveals that for adult females, separation of the sibling group is so distinctive that the AQ scores of low‐scoring siblings do not differ from control participants, while high‐scoring siblings closely resemble the ASC group, even though these siblings did not have a clinical diagnosis. In summary, this suggests that both high and low scoring adult female siblings have relatively more autistic traits than their counterparts in the other groups. There may be a difference in BAP for females. Alternatively, it is possible that this reflects female siblings being under‐diagnosed. Future work could test whether members of this group have gone on to receive a diagnosis of ASC, or meet research diagnostic criteria for ASC using standardized measures such as the ADOS/ADI‐R.

### Limitations

A limitation to a large online study of this kind is about the reliability of the diagnostic information. This risk was minimized by asking for details of the date of diagnosis, and the clinic, and restricting the sample to those who had received a DSM‐IV/5 diagnosis from a clinical psychologist or psychiatrist. Online reporting of diagnosis has been shown to be highly reliable [Lee et al., 2010]. Further, in investigating the BAP in siblings, we acknowledge that many of the individuals we would like to include in research studies may fall into a “blurry” category populated by individuals who may meet the diagnostic criteria for ASC but who have not received a diagnosis. Another limitation is that it is difficult to control variables such as sibling pair gender dyads and birth order, which may influence behavior and ratings on measures of autistic traits. For instance, families where all siblings are the same gender have been shown to be less influenced by gender stereotypes [Anelli & Peri, 2015]. It has also been shown that shared environment, as well as genetic relatedness, may also play a role in ASC [Hallmayer et al., 2011]. The current study design lacked sufficient statistical power to test these factors, but future studies should investigate to see the effect of these factors. In addition, the AQ is a self‐report or parent‐report measure that relies on the perception of autistic traits by individuals. Future research might test our findings by accompanying the AQ with a diagnostic assessment, such as the Autism Diagnostic Observation Schedule (ADOS) [Lord et al., 1989].
